# Multiparameter antigen-specific immunoprofiling in subjects with negative IGRA and TST results with potential *M. tuberculosis* exposures

**DOI:** 10.3389/fcimb.2026.1837269

**Published:** 2026-05-01

**Authors:** Balaji Pathakumari, Luis E. Gutierrez, Shaji Ahmed Faisal, Pedro Arias Sanchez, Kale Daniel, Virginia P. Van Keulen, Heather R. Hilgart, Manik R. Reddy, Elitza Theel, Rafael Laniado-Laborin, Jeremy M. Clain, Tobias Peikert, Ryan C. Bailey, Patricio Escalante

**Affiliations:** 1 Division of Pulmonary, Critical Care, Allergy and Sleep Medicine, Department of Medicine, Mayo Clinic, Rochester, MN, United States; 2 Department of Laboratory Medicine, Mayo Clinic, Rochester, MN, United States; 3 Department of Chemistry, University of Michigan, Ann Arbor, MI, United States; 4 Clinica y Laboratorio de Tuberculosis, Facultad de Medicina y Psicologia, Hospital General Tijuana, Universidad Autonoma de Baja California, ISESALUD, Tijuana, Baja California, Mexico

**Keywords:** latent tuberculosis infection, tuberculosis exposure, flow cytometry, TST/IGRA, T cell markers

## Abstract

**Introduction:**

Asymptomatic individuals with negative Interferon-γ release assay (IGRA) and tuberculin skin test (TST) results are often labeled as tuberculosis-unexposed individuals. We hypothesize that flow cytometric (FC) detection of antigen-stimulated T-cells in peripheral blood can differentiate IGRA(-)/TST(-) individuals with and without prior potential *M. tuberculosis* (Mtb) exposure.

**Methods:**

We used FC multiparameter profiling of T-cell activation markers in TST/IGRA-negative individuals at risk for Mtb exposure and in unexposed controls.

**Results:**

We studied 54 IGRA(-)/TST(-) subjects, including 27 individuals at risk for prior Mtb exposure. While quantitative IGRA results were negative in all patients, FC profiling of CD3/CD4^+^IFN-γ^+^HLA-DR^+^ T-cells after stimulation with a pool of Mtb-specific region of difference-1 (RD1) peptides, mycobacterial (PPD and MTB300), or *Candida* antigens, statistically differentiated between unexposed and potentially exposed individuals (P<0.05). ROC analysis showed PPD and RD1 peptides responses had AUCs >0.7 (sensitivity of 55.5–74% and specificity of 66.6–85.1%). The presence of a polyfunctional RD1-reactive CD3/CD8^+^IFN-γ^+^TNF-α^+^ T-cell subset also significantly differentiated these groups (AUC 0.66). Other T-cell markers demonstrated no significant differences.

**Conclusions:**

FC detection of antigen-specific IFN-γ^+^HLA-DR^+^ CD4+T-cells and IFN-γ^+^TNF-α^+^ CD8^+^ T-cells can differentiate IGRA(-)/TST(-) individuals at risk of Mtb exposure from unexposed controls. Further validation is needed to determine whether this immunoprofiling approach can improve detection of Mtb infections and identification of unexposed individuals for future biomarker studies and vaccine trails.

## 1 Introduction

Tuberculosis (TB) remains a critical global health challenge, with latent TB infection (LTBI) estimated to affect about one quarter of the world’s population (Cohen et al., 2019). In the United States, the true prevalence of LTBI remains unknown, but current estimates suggest that approximately 5% of the population may harbor LTBI (Force et al., 2023). Early identification of individuals at risk for TB is essential to enable timely intervention and prevent disease transmission. Screening for LTBI in individuals at high risk is a cornerstone of the World Health Organization’s End TB strategy, where targeted preventive treatment can significantly reduce progression to active disease (Barry et al., 2009; Goletti et al., 2025; Mangione et al., 2023). However, detecting *M. tuberculosis* (Mtb) infection during latent and asymptomatic phases remains a major challenge (Escalante and Wilson, 2021). This difficulty is more problematic among individuals who test negative with the available clinical tests for LTBI diagnostic assessments, including both the Interferon-Gamma Release Assay (IGRA) and the Tuberculin Skin Test (TST). In clinical and research contexts, asymptomatic individuals with low TB exposure risk and negative TST/IGRA results are typically classified as unexposed or uninfected. However, these tests cannot reliably detect individuals who have been exposed to Mtb but remain uninfected (Herrera et al., 2011). These exposed individuals with negative TST/IGRA results may have achieved Mtb clearance prior to mounting adaptive immune responses, or they may harbor dormant Mtb not detectable by IGRA or TST methods. The ability to identify exposed individuals would greatly enhance TB diagnostics and epidemiological research, as well as TB vaccine trial cohort selections. As such, there is an urgent need for specific biomarkers capable of distinguishing between Mtb exposed and unexposed individuals.

Recent studies have shown that antigen-specific HLA-DR co-expression of IFN-γ and TNF-α on CD3+ T cells can differentiate individuals with recent or prior Mtb exposure from those without, as well as distinguish LTBI from active TB (Luo et al., 2021; Mpande et al., 2021a). Earlier, we have demonstrated that combinatorial antigen-specific immune profiling using T-cell activation markers has potential for refining TB risk and exposure assessment (Escalante et al., 2015). The application of HLA-DR and other T-cell activation markers has therefore emerged as a promising approach for identifying individuals who may benefit from further clinical evaluation, enabling more precise immunoprofiling stratification of LTBI.

In this study, we aimed to test a multiparameter immunoprofiling approach designed to detect antigen-specific activated T-cell subsets potentially capable of distinguishing IGRA-negative/TST-negative individuals previously exposed to Mtb from unexposed individuals. Specifically, we utilized previously described immunoprofiling methods that measure antigen-specific T-cell activation with co-expression of HLA-DR and intracellular cytokines such as IFN-γ and TNF-α as well as activation-induced markers on T-cells such as CD25, CD134, and PD-L1. By utilizing these immunoprofiling methodologies, our main study goal is to improve the accuracy of Mtb exposure assessment and contribute to the development of more effective TB control and prevention strategies.

## 2 Materials and methods

### 2.1 Study participants

The study was approved by the Mayo Clinic Institutional Review Board and Olmsted County Public Health Services (IRB 09–003253). All participants provided written informed consent and were enrolled at Mayo Clinic, Rochester, Minnesota, between September 2020 and August 2024. All study procedures were conducted in accordance with relevant guidelines and regulations, following approval and recommendations from the Mayo Clinic IRB.

This research was part of a prospective, single-center, laboratory technician-blinded study investigating new immune biomarkers in tuberculosis. The study involved consecutive enrollment of age- and sex-matched TB-unexposed controls and subjects with a history of potential Mtb exposure. Potentially TB-exposed subjects included individuals with close contact with active TB cases, individuals who migrated from TB endemic regions, healthcare workers with direct patient care, and laboratory personnel at potential risk of exposure. TST testing was performed according to national guidelines, with results retrieved from electronic medical records (EMR). Study inclusion criteria for potential TB-exposed individuals also included being asymptomatic, and with negative clinical and radiological signs of active TB, along with a lack of evidence of Mtb infection by available clinical laboratory tests. TB-unexposed individuals were defined as US-born people or individuals born in other low-TB endemic areas with no known history of close contact with active TB cases, no recent travel to or residence in high TB endemic countries, and no occupational or environmental risk factors for Mtb exposure.

### 2.2 Interferon-gamma release assay

Tuberculosis infection status was confirmed using the fourth-generation QuantiFERON TB gold plus (QFT-Plus) assay (Qiagen™, Hilden, Germany), which quantifies IFN-γ concentrations in response to Mtb-specific antigens (ESAT-6, CFP-10) in two tubes (TB1 and TB2). We used automated chemiluminescent immunoassay (CLIA; LIAISON XL, DiaSorin) with confirmatory ELISA (Qiagen) when necessary, applying the standard positivity cutoff of ≥0.35 IU/mL (TB1-nil or TB2-nil) for both platforms.

### 2.3 PBMC isolation

Peripheral blood mononuclear cells (PBMCs) were isolated from sodium-heparinized blood using Ficoll-Paque PLUS density gradient centrifugation (Cytiva, Uppsala, Sweden) at 400×g for 30 min. Viable cell counts were determined via trypan blue exclusion on a hemocytometer. Cells were cryopreserved in freezing medium (10% DMSO/90% cosmic calf serum; Fisher Scientific) and stored in liquid nitrogen for downstream immunoprofiling.

### 2.4 Flow cytometry

Cryopreserved PBMCs were thawed and cultured in RPMI 1640 medium (Sigma-Aldrich, St. Louis, MO) supplemented with 10% fetal bovine serum. Cells were stimulated with costimulatory antibodies (CD28 and CD49d, 1 μg/mL each; BD Biosciences, San Diego, CA) and a panel of test antigens, including ESAT-6 (4 μg/mL)/CFP-10 (2 μg/mL) (RD1-region of difference) peptide pools (GenScript Biotech, Piscataway, NJ, USA), purified protein derivative (PPD) (10 μg/mL; AJ Vaccines, Copenhagen, Denmark), MTB300 peptide pool (0.5 μg/mL); courtesy from Dr. Lindestam-Arlehamn), *Candida albicans* native protein (*Candida* antigen) (2 μg/mL; MyBioSource, San Diego, CA, USA), and phytohemagglutinin (PHA) (0.2 μg/mL; Thermo Fisher Scientific, MA, USA) Unstimulated cells cultured under identical conditions served as negative controls. PBMCs were incubated with antigens or controls for 18 hours, with Brefeldin A (Sigma) added during the final 4 hours provided the balance between maintaining PBMC viability and detecting intracellular cytokine and surface markers.

Following stimulation, cells were stained with anti-human monoclonal antibodies targeting surface markers: CD3 (488B), CD4 (APC.H7), HLA-DR (PE) (BD Biosciences, San Diego, CA) CD8 (BV510), CD134 (BV420), PD-L1 (BV605), and CD25 (AF700) (BioLegend, San Diego, CA) along with live/dead fixable dye (ThermoFisher Scientific, Waltham, MA). Intracellular cytokine staining for IFN-γ (APC) and TNF-α (PE.Cy7) (BD Biosciences, San Diego, CA) was then performed. After staining, cells were washed, fixed with 0.5% paraformaldehyde. A minimum of 250,000 events was acquired per sample using a BD LSRFortessa flow cytometer (BD Biosciences, San Diego, CA). Data were exported in FCS 3.0 format and analyzed with Kaluza software (Beckman Coulter, Brea, CA). The frequency of phenotypic markers was determined after subtracting background responses from unstimulated controls. The general gating strategy for identifying CD4^+^ T cells expressing HLA-DR and IFN-γ is illustrated in **Supplementary Figure 1**.

### 2.5 Data analysis

Categorical variables were compared using Chi-square and Fisher’s exact test. Data are presented as counts and percentages or as medians with interquartile ranges (IQR), as appropriate. Group comparisons for continuous variables were performed using the non-parametric Mann–Whitney U-test. Diagnostic performance of immune markers was assessed using receiver operating characteristic (ROC) curve analysis, with area under the curve (AUC), sensitivity, and specificity calculated at 95% confidence intervals (CI) to differentiate study groups. Statistical significance was set at p < 0.05. All analyses were conducted using GraphPad Prism 9.3.1 (GraphPad Software, San Diego, CA).

## 3 Results

### 3.1 Baseline characteristics

This study included 54 participants who tested negative for both IGRA/TST, comprising 27 individuals identified as being at risk of prior potential Mtb exposure. The demographic characteristics of the study groups are summarized in **Table 1**. A higher proportion of the Mtb exposed group were born outside the United States (18.5% from high TB-incidence countries), 2 with prior BCG vaccination (7.4%), and 21 healthcare workers (HCWs) with direct patient care (77.8%). By design, none of the unexposed controls had these exposure-risk characteristics. The groups were well-matched for age (10-year range) and sex and thus, no statistically significant differences were observed in these characteristics across these two groups.

**Table 1:** Characteristics of study subjects.

Subjects Characteristics	All subjects n=54	Unexposed n=27	Potential TB exposure(s)^a^ n=27	P value^b^
Male sex	22 (40.7)	11 (40.7)	11 (40.7)	>0.999
Age, years				
Mean ± SD	41.04 ± 14.89	40.96 ± 14.25	41.11 ± 15.80	0.971
Range	21-82	21-76	21-82	
Ethnicity				0.107
Caucasian	48 (88.8)	26 (96.2)	22 (81.4)	
African American	3 (5.2)	1 (3.7)	2 (7.4)	
Asian Pacific	0 (0)	0 (0)	0 (0)	
Hispanics	2 (3.5)	0 (0)	2 (7.4)	
Others	1 (1.8)	0 (0)	1 (3.7)	
Place of Birth^c^				0.051
US Born	49 (90.7)	27 (100)	22 (81.5)	
Foreign Born (High TB)	5 (9.2)	0 (0)	5 (18.5)	
Foreign Born (Low TB)	0 (0)	0 (0)	0 (0)	
BCG vaccination				0.038
Yes	2 (3.7)	0 (0)	2 (7.4)	
No	46 (85.1)	26 (96.2)	20 (74)	
Unknown	6 (11.1)	1 (3.7)	5 (18.5)	
History of HIV infection				>0.999
HIV (+ve)	1 (1.8)	0 (0)	1 (3.7)	
HIV (-ve)	30 (55.5)	15 (55.5)	15 (55.5)	
Unknown	23 (42.5)	12 (44.4)	11 (40.7)	
Occupation				<0.001
HCW, direct patient care	21 (38.8)	0 (0)	21 (77.7)	
HCW, no direct patient care	0 (0)	0 (0)	0 (0)	
Other	33 (61.1)	27 (0)	6 (22.2)	
TB contact or potential exposure(s) time				<0.001
Recent (<5y)	8 (14.8)	0 (0)	8 (29.6)	
Remote (>5y)	5 (9.2)	0 (0)	5 (18.5)	
Unknown	14 (25.9)	0 (0)	14 (51.8)	
QFT result				1.000
Positive	0 (0)	0 (0)	0 (0)	
Negative	54 (100)	27 (100)	27 (100)	
TST result				0.2501
Positive	0 (0)	0 (0)	0 (0)	
Negative	36 (66.6)	15 (55.5)	21 (77.7)	
Unknown	18 (33.3)	12 (44.4)	6 (22.3)	

IGRA, Interferon Gamma Release Assay; TST, Tuberculin Skin Test; BCG, Bacillus Calmette-Guerin; HIV, human immunodeficiency virus; HCW, Health Care Worker.

a. TB exposure includes the history of contact with active TB cases and HCWs with direct patient contact.

b. P values were derived from non-parametric test comparison between unexposed and Mtb exposed groups.

c. Incidence rate is ≥40 cases per 100,000 people defines high incidence of tuberculosis.

### 3.2 TB1 and TB2 IFN-γ responses in QFT-Plus

**Figure 1:**
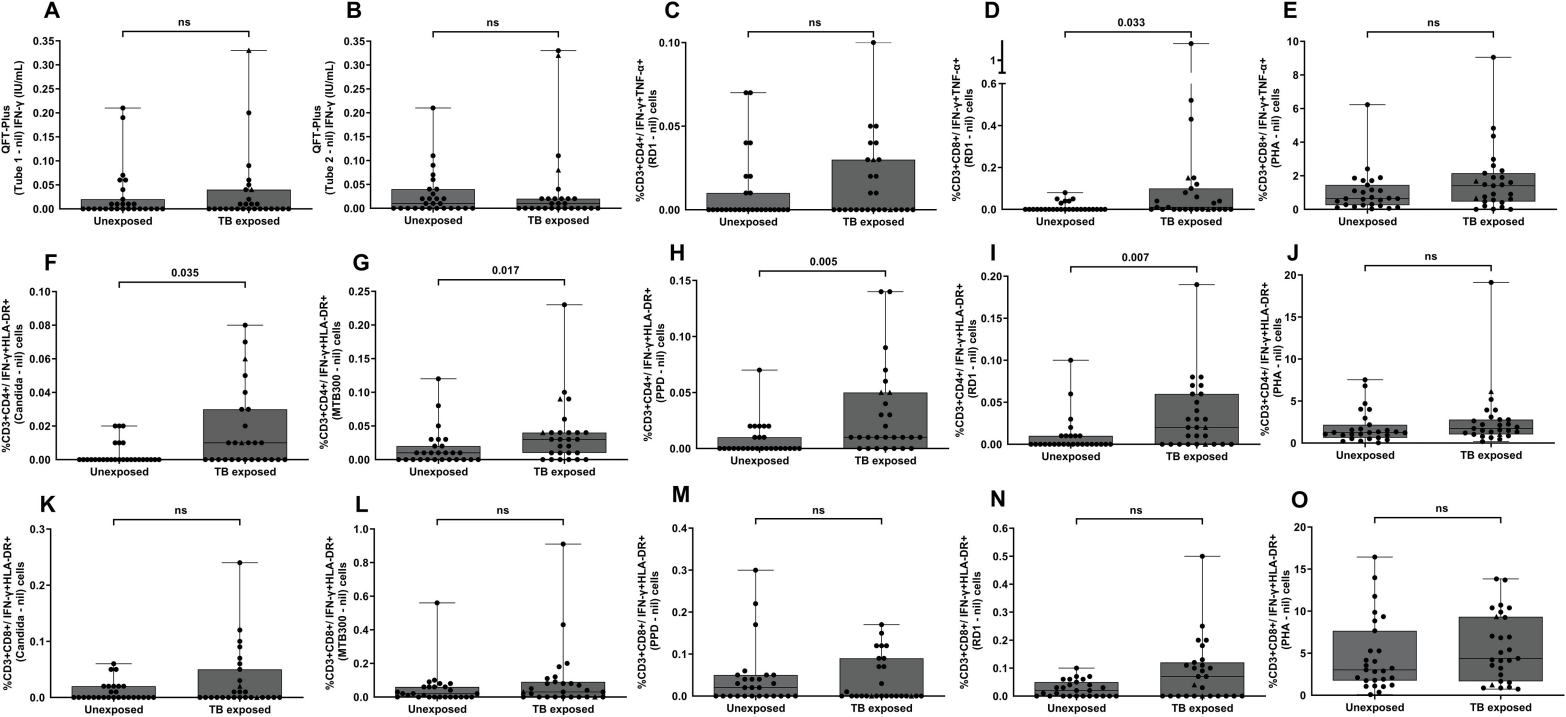
Functional profile of QFT-Plus and FC assays in TB unexposed and exposed groups. QFT-Plus and the frequency of FC T-cell subsets in TB unexposed and exposed individuals. **(A)** QFT-Plus (tube 1-nil), **(B)** QFT-Plus (tube 2-nil). The percentage of RD1 specific CD4+ secreting IFN-γ+TNF-α+ **(C)**, CD8+ secreting IFN-γ+TNF-α+ against RD1 **(D)** and PHA **(E)**. The middle panel shows the percentage of CD4^+^ T cells co-expressing IFN-γ^+^HLA-DR^+^ in response to Candida antigen **(F)**, MTB300 **(G)**, PPD **(H)**, RD1 peptides **(I)**, PHA **(J)**. The lower panel indicates the percentage of CD8^+^ T cells co-expressing IFN-γ^+^HLA-DR^+^ in response to Candida antigen **(K)**, MTB300 **(L)**, PPD **(M)**, RD1 peptides **(N)**, PHA **(O)**. For each donor, responses to stimulated cells were background-subtracted. Two BCG-vaccinated TB-exposed individuals are marked with triangle shapes on the plot to distinguish them from non-vaccinated subjects. Differences between groups were compared using the Mann–Whitney U-test. ns = not significant (P ≥ 0.05). The horizontal line represents the median; the upper and lower boundaries of each box represent the 75th and 25th percentiles, respectively. Whiskers extend to the minimum and maximum values.

Both mean TB1 and TB2 (both minus nil) IFN-γ responses were higher but not statistically different in the TB-exposed group compared to unexposed controls (**Supplementary Table 1**). The mean TB1 IFN-γ response in the TB-exposed group was 0.032 IU/mL, while the unexposed group had a mean of 0.025 IU/mL. Similarly, the mean TB2 IFN-γ response was 0.038 IU/mL in the TB-exposed group and 0.028 IU/mL in the unexposed group (**Figure 1**). These findings suggest that quantitative IFN-γ responses measured by QFT-Plus alone may lack sufficient sensitivity to reliably identify Mtb exposure in this cohort.

### 3.3 Antigen-specific T-cell activation markers in subjects with potential Mtb exposure versus unexposed individuals

**Figure 2:**
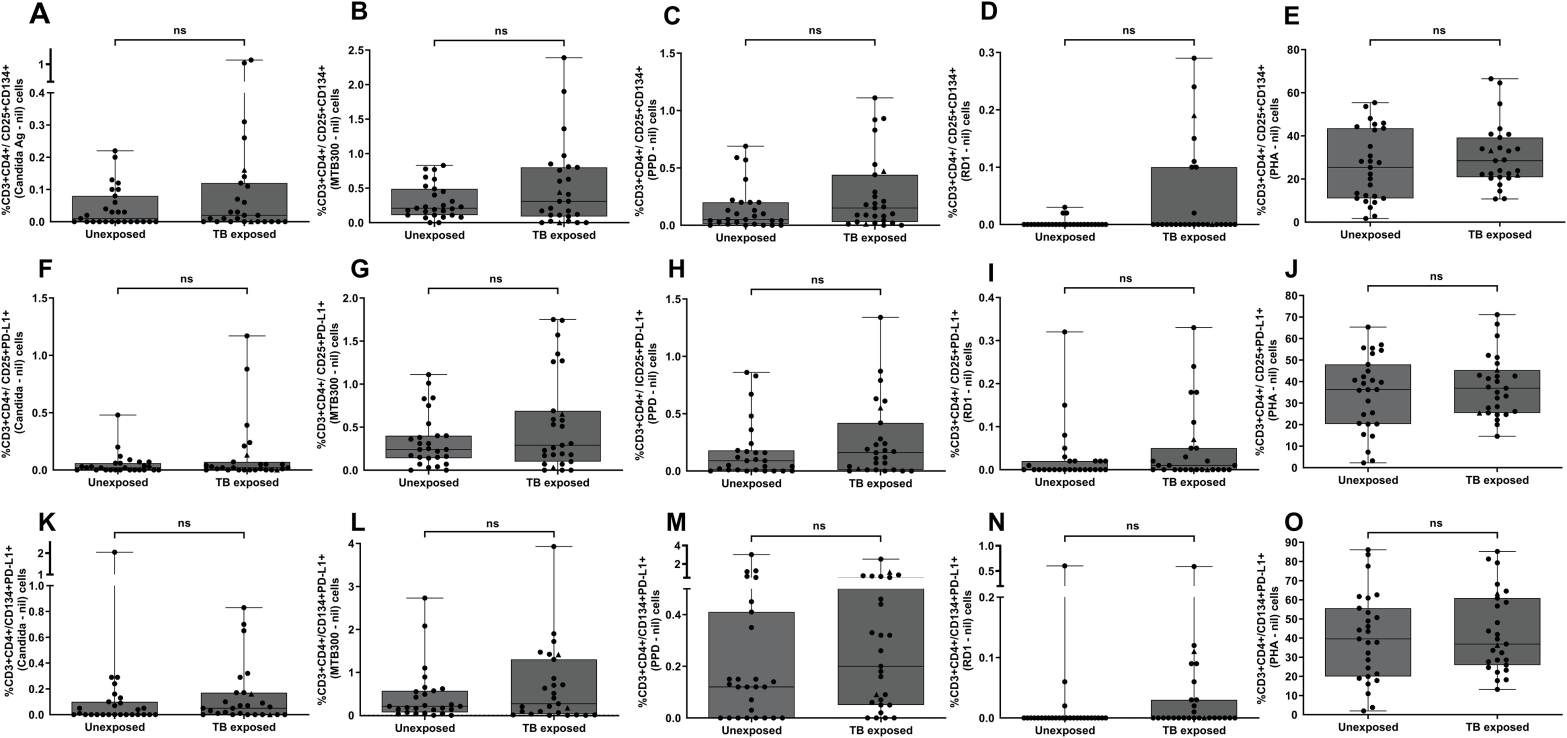
Immunophenotyping results of FC assays in TB unexposed and exposed groups. Flow cytometric detection of the percentage of CD3^+^CD4^+^ T cells co-expressing CD25^+^CD134^+^ (upper panel: **A–E**), CD25^+^PD-L1^+^ (middle panel: **F–J**), and CD134^+^PD-L1^+^ (lower panel: **K–O**) following stimulation with Candida antigen, MTB300, PPD, RD1 peptides, and PHA. For each donor, responses to stimulated cells were background-subtracted. Two BCG-vaccinated TB-exposed individuals are marked with triangle shapes on the plot to distinguish them from non-vaccinated subjects. Group differences were analyzed using the Mann–Whitney U-test. The horizontal line indicates the median; the upper and lower boundaries of each box represent the 75th and 25th percentiles, respectively. Whiskers extend to the minimum and maximum values. ns = not significant (P ≥0.05).

**Figure 3:**
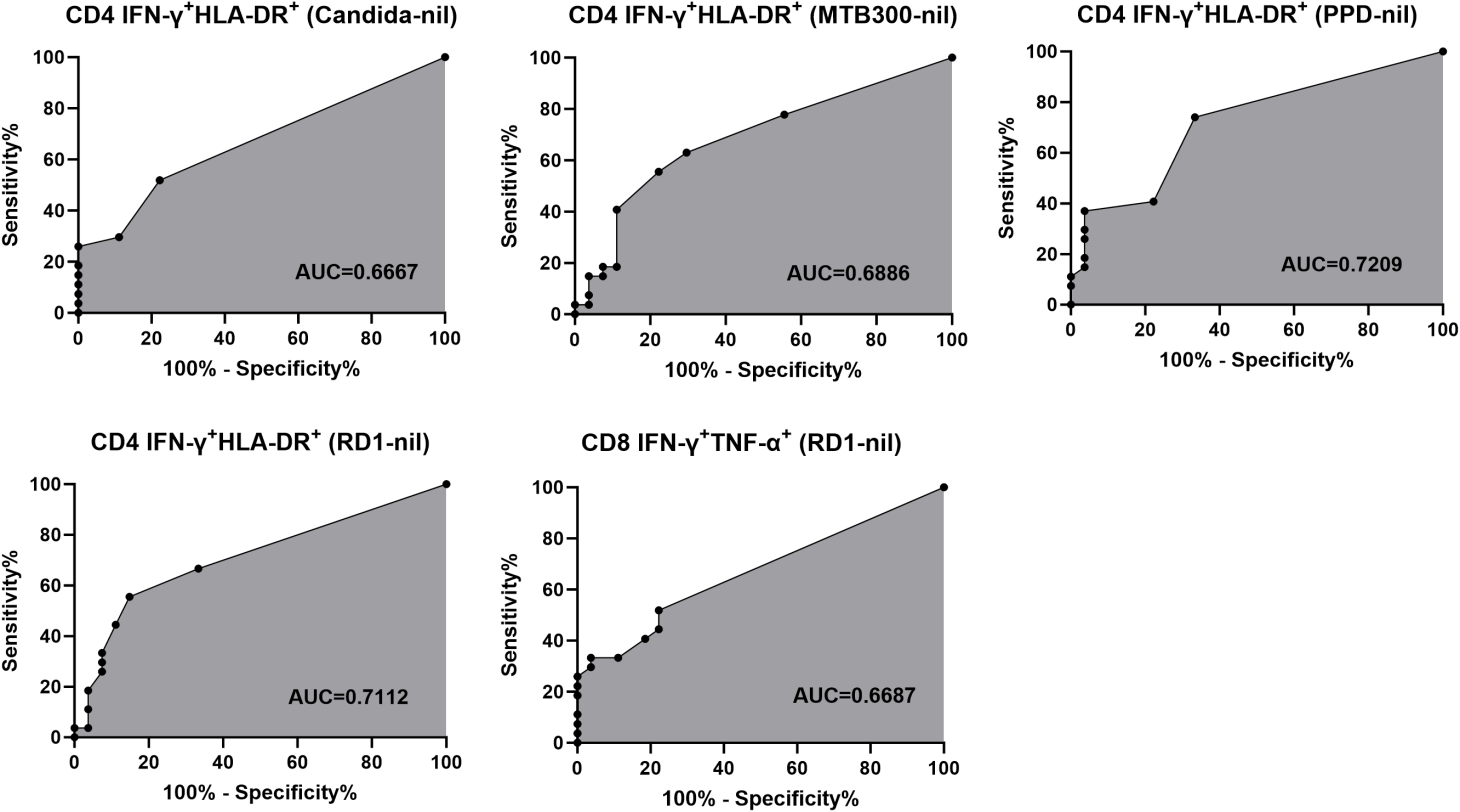
ROC curve analysis for antigen-specific T-cell activation markers in distinguishing TB-exposed from unexposed individuals. Receiver operating characteristics curve (ROC) plots show the diagnostic accuracy of CD4^+^ T cells co-expressing HLA-DR and IFN-γ and dual functional CD8 secreting IFN-γ and TNF-α after stimulation with Candida antigen, MTB300, PPD, and RD1 peptides. The area under the curve (AUC) quantifies the overall diagnostic performance. Data points represent the balance between true positive and false positive rates for various thresholds. All analyses were performed using background-subtracted frequencies for each donor.

The frequency of CD3^+^CD4^+^IFN-γ^+^HLA-DR^+^ T cells was significantly higher in potentially TB-exposed individuals than in unexposed controls following stimulation with RD1 peptides, MTB300, PPD, and *Candida* antigens (p < 0.05; **Figure 1**). In contrast, no significant differences were observed between groups for other CD4+T-cell intracellular (TNF-α+HLA-DR+), and surface markers, such as CD25^+^CD134^+^, CD25^+^PD-L1^+^, and CD134^+^PD-L1^+^, although an upward trend was noted for MTB300 and PPD in the TB-exposed group (**Figure 2**, and **Supplementary Table 1**). Receiver Operating Characteristic (ROC) curve analysis demonstrated that both RD1 and PPD stimulations provided good discriminatory power, with AUC > 0.7 (**Figure 3**). Specifically, RD1 stimulation yielded a sensitivity of 55.5% (95% CI: 37.3–72.4) and a specificity of 85.1% (95% CI: 67.5–94.0) with this T-cell markers, while PPD demonstrated a higher sensitivity of 74% (95% CI: 55.3–86.8) but a lower specificity of 66.6% (95% CI: 47.8–81.3) (**Table 2**). IFN-γ^+^HLA-DR^+^ in CD4^+^ T cells after MTB300 and *Candida* antigen stimulations also differentiated TB-exposed from unexposed individuals (AUCs of 0.68 and 0.66, respectively; **Figure 3**). MTB300 peptide stimulation showed a sensitivity similar to RD1 but a lower specificity of 77.7% (95% CI: 59.2–89.3). *Candida* antigen stimulation, as a nonspecific antigen, had comparable specificity to MTB300 antigen response but reduced sensitivity (51.8%) (**Table 2**). Additionally, polyfunctional RD1-reactive CD3^+^CD8^+^ T cells co-expressing IFN-γ and TNF-α provided moderate but significant differentiation (p = 0.033 and AUC of 0.66), with sensitivity of 51.8% (95% CI: 33.9–69.2), and specificity of 77.7% (95% CI: 59.2–89.3) (**Table 2**).

**Table 2:** Diagnostic potential of functional activation index in TB unexposed and exposed subjects.

Phenotype	Sensitivity (%)	Specificity (%)	Cut-off
CD3^+^CD4^+^IFN-γ^+^HLA-DR^+^ (Candida -nil)	51.8 (95 CI: 33.9-69.2	77.7 (95 CI: 59.2-89.3)	>0.005%
CD3^+^CD4^+^IFN-γ^+^HLA-DR^+^ (MTB300 -nil)	55.5 (95 CI: 37.3-72.4)	77.7 (95 CI: 59.2-89.3)	>0.025%
CD3^+^CD4^+^IFN-γ^+^HLA-DR^+^ (PPD -nil)	74.0 (95 CI 55.3-86.8)	66.6 (95 CI: 47.8-81.3)	>0.005%
CD3^+^CD4^+^IFN-γ^+^HLA-DR^+^ (RD1 -nil)	55.5 (95 CI: 37.3-72.4)	85.1 (95 CI:67.5-94.0)	>0.015%
CD3^+^CD8^+^IFN-γ^+^TNF-α^+^ (RD1 -nil)	51.8 (95 CI: 33.9-69.2)	77.7 (95 CI: 59.2-89.3)	>0.005%

We further conducted an exploratory subgroup analysis stratifying TB−exposed donors into recent (<5 years, n=8) and remote (>5 years, n=5) exposure. In this subgroup analysis, we did not observe statistically significant differences in functional T−cell frequencies by antigen-specific IFN-γ^+^HLA-DR^+^ CD4^+^ T cells profiling between these subgroups. However, we observed significant differences in the percentage of RD1−specific CD4^+^CD25^+^PD−L1^+^ and CD4^+^CD134^+^PD−L1^+^ T cell activation (**Supplementary Figure 2**).

In addition to standard background subtraction, we normalized our datasets by calculating the ratio of activated antigen-specific cells (CD4^+^/CD8^+^IFN-γ^+^HLA-DR^+^) to total antigen-specific cells (CD4^+^/CD8^+^IFN-γ^+^) as shown to differentiate subjects with recent IGRA conversions from individual with persistent IGRA positivity in an endemic TB area (Mpande et al., 2021a). This type of immunoprofiling normalization approach showed no statistically significant differences between unexposed and TB-exposed groups for any tested antigens (**Supplementary Figure 3**). Even though these ratios can be informative, they can be also more sensitive to changes by small denominator numbers and probably less stable for longitudinal measurements and diagnostic classification than absolute or background−subtracted frequencies (Maecker et al., 2005; Jelliffe et al., 2015; Mizrahi et al., 2018).

## 4 Discussion

Our exploratory study results suggest that a significant number of individuals with history or potential Mtb exposure, and with negative TST/IGRA results exhibit a significantly higher percentage of CD3/CD4^+^IFN-γ^+^HLA-DR^+^ cells following stimulation with RD1 peptides, MTB300, and PPD, compared to unexposed controls, highlighting the activation of antigen-specific T cell responses upon mycobacterial antigenic challenge. These antigen-specific T cell subset findings probably suggest the development of immunological memory or priming in individuals with prior Mtb exposure(s), consistent with previous reports (Mpande et al., 2021a, 2021b).

This is an important study finding since approximately 50% of individuals with close TB exposures remain persistently negative on TST and IGRA testing Mtb (Morrison et al., 2008). This relatively common scenario has led to the hypothesis that some TB-exposed individuals may be “resistor” to Mtb infection through innate or other immune mechanisms but fail to acquire adaptative memory immune responses and thus, cannot be detected by our available clinical laboratory assays or TST (Barry et al., 2009; Pai, 2010; Luo et al., 2021). Epithelial sensing, rapid activation of innate immune cells, autophagy, early cytokine release, and favorable genetic factors have been postulated to be involved in Mtb clearance before mounting adaptive immune responses after Mtb exposure (Simmons et al., 2018). As a result, there is a potential risk of underestimation of detecting a subset of TB-exposed individuals with our available diagnostic methods, which is an important gap in the field.

Of note, individuals with negative TST and IGRA results cannot be reliably classified as Mtb-uninfected, post−clearance, or currently infected, because both assays have imperfect accuracy and some patients with active TB can also have negative IGRA/TST results (Pai, 2010). Importantly, our study subjects were thoroughly evaluated at our referral medical center, and active TB was excluded on clinical, radiological, and microbiological grounds according to current guidelines, and thus, no active TB cases were included. In this context, it is possible that our study group of TB-exposed and TST/IGRA−negative individuals may still harbor undetected Mtb infection or have detectable memory T-cell responses after clearing Mtb infections. Moreover, our multiparametric antigen-specific assay method that utilizes co-stimulation reagents might be able to detect antigen-specific cellular responses that our clinical laboratory methods might not in some individuals. In addition, QFT−Plus IFN−γ responses to TB1 and TB2 (antigen minus nil) in our TB-exposed study group were at very low concentrations and far below the diagnostic cutoff thresholds (mean TB1 0.032 IU/mL, TB2 0.038 IU/mL), making false−negative IGRA results unlikely. The positive responses in our FC immunoprofiling results in these IGRA/TST negative individuals reveals that the immune response is not captured by standard testing like IGRA. However, the underlying biological state remains uncertain and requires additional investigations, including longitudinal studies.

This diagnostic uncertainty highlights the need for more accurate biomarkers and diagnostic methods to reliably identify and characterize individuals who resist or clear Mtb infection after TB exposure(s) or have undetectable Mtb infections with our available diagnostic methods. In Uganda, 10–21% of highly exposed household TB contacts remained TST/IGRA-negative over 1–9.5 years, indicating persistent lack of TST/IGRA detectable immune conversion or lack of mounting an adaptive immune response despite TB exposure(s) (Lu et al., 2019). Similarly, a multinational study found that 10–13% of household contacts showed “resistance” to Mtb infection when using strict TST diagnostic cutoffs of <5 mm or 0 mm were applied (Baliashvili et al., 2021). In addition, up to 8.4% of healthcare workers in high-endemic TB settings remained persistently TST/IGRA-negative despite occupational Mtb exposure(s), reinforcing the observation of “TB resistors” across different high-risk groups (Lu et al., 2019). This evidence from high-endemic TB settings also suggests a substantial gap in our ability to accurately and reliably identify individuals with actual Mtb exposure with the available diagnostic methods, which could also limit the strict selection of unexposed individuals for TB vaccine trials. These findings underscore an important need to improve diagnostics for Mtb exposed individuals to better detect and characterize Mtb exposures and Mtb infections for public health, clinical, and investigational purposes.

The marginal difference in mean TB1 and TB2 IFN-γ responses in our cohort highlights the limited sensitivity of quantitative IGRA responses. Furthermore, the clinical utility of the TB2–TB1 difference as a proxy for CD8+ T-cell response or recent Mtb infection has been questioned, as studies have not found a consistent association between higher TB2 responses and increased risk of recent Mtb exposure or disease progression (Yi et al., 2016; Perez-Recio et al., 2021). In our study cohort, TB1 and TB2 responses also did not significantly differentiate potentially TB-exposed individuals from unexposed subjects.

Our study results also suggest that FC antigen-specific T-cell activation by multiparameter profiling is a promising method for early and accurate detection of TB exposures; however, standardization of these functional immunoprofiling methods across laboratories can be challenging and subject to similar technical limitations of other clinical immunoassays (Halliday et al., 2017; Mpande et al., 2021a, 2021b). Previous studies have demonstrated that T cells co-expression of HLA-DR and IFN-γ after Mtb-antigen stimulations are upregulated in individuals with recent Mtb infection and high likelihood to progress to TB disease. In contrast, those with remote or nonprogressive Mtb infection exhibit lower activation of these antigen-specific T cell markers (Mpande et al., 2021a).

Mtb-specific HLA-DR upregulation on IFN-γ–producing T cells demonstrated strong diagnostic potential, with substantial sensitivity and specificity for distinguishing TB-exposed from unexposed individuals, suggesting an additional value in TB immunodiagnostics. Moreover, our FC immunoprofiling approach directly measures antigen-specific T cell activation, providing greater specificity for distinguishing TB-exposed individuals and suggesting that FC-based immunodiagnostic applications can provide important additional TB exposure information compared to TST and IGRA (Leung et al., 2009; Papageorgiou et al., 2016; Mpande et al., 2021a). Other, less Mtb-specific antigens, such as PPD and MTB300 showed lower diagnostic accuracy to differentiate individuals with history of TB exposure or potential exposure from unexposed subjects. These immunoprofiling study results are likely associated with sharing epitopes of Mtb with other mycobacteria and possibly cross-reactivity with non-tuberculosis exposures in our unexposed study group. PPD and MTB300 can also elicit T-cell response in BCG-vaccinated individuals and patients with NTM infections due to antigenic overlap (Lindestam Arlehamn et al., 2015). In our cohort, prior BCG vaccination was reported in only 2 out of 27 TB-exposed individuals (7.4%) and thus, this minor imbalance in the group comparison is unlikely to alter our immunoprofiling results. Moreover, immune cross-reactivity with PPD is much more attenuated after 15 years post-BCG vaccination and thus these 2 previously BCG-vaccinated adult participants were unlikely to be the primary drivers of the observed study group differences (**Figure 2**, **3**) (Mancuso et al., 2017).

Interestingly, our TB-exposed study group also showed significant upregulation in IFN-γ^+^HLA-DR^+^ responses to Candida antigen. The explanation for these immunoprofiling results is unclear and we can only speculate that these are associated with heterologous immunity, where recent Mtb exposure generates cross-reactive T cells or boosts general Th1 responsiveness to these apparently unrelated organisms. However, we previously observed similar heterologous responses to candida antigen with ELISpot and flow cytometry assays, though this finding requires further investigation (Marty et al., 2024). Furthermore, the polyfunctional profile of RD1-reactive CD3^+^CD8^+^ T cells co-expressing IFN-γ and TNF-α, can also differentiate potentially Mtb exposed from unexposed individuals. Notably, no significant differences were observed in the expression of T-cell surface phenotypic markers, despite an upward trend in PD-L1 expression in T cells after PPD and MTB300 stimulations in the TB-exposed study group, which can provide important insights into immune regulation after Mtb exposure (Lindestam Arlehamn et al., 2015; Tezera et al., 2020).

Previous studies have shown that T-cell markers such as CD38, Ki-67, and PD-1 can serve as early immunological indicators of recent Mtb exposure, even before IGRA conversion (Diaz-Fernandez et al., 2022). However, most of our study subjects were not recently exposed, and it remains to be seen whether our immunoprofiling approach can also detect earlier stages of Mtb exposure, which could offer valuable insights into the dynamics of host immune activation during early stages of Mtb infections. Another important observation is that some of the potentially Mtb exposed individuals showed no detectable antigen-specific response. These individuals likely represent epidemiologically and biologically heterogeneous group but clinically relevant from scientific and practice perspective. It is possible that some of these “Potentially Mtb-exposed” individuals were not actually exposed to Mtb. Potential biological explanations can include remote Mtb exposure with waning peripheral immune responses, due to natural attrition of memory T-cell clones in circulation over time, true innate immune resistance or early clearance after Mtb exposures, inter-individual variability in T-cell activation. Moreover, these immune responses were not identical across all antigens, suggesting only partial overlap among these individuals. Our study was exploratory and results require further validations, but if this immunoprofiling results hold true, this method can assist in further characterize and study “TB resistors” who remain persistently TST- and IGRA-negative despite high Mtb exposures (Verrall et al., 2014). Identifying and characterizing these “TB resistors” is essential for selecting unexposed subjects for TB vaccine studies and elucidating host-pathogen interactions associated with natural protection (Zhuang et al., 2023). These findings highlight the importance of accurately monitoring temporal immune dynamics to distinguish true Mtb infection from immune clearance or resistance, advancing both TB diagnostics and our broader understanding of protective immunity (Verrall et al., 2014; Ding et al., 2025).

The strengths of our study include immunoprofiling of well-matched cohorts by age and sex, minimizing these potential confounders, and assessment of cellular immune competence through robust mitogen PHA control conditions. Although the diagnostic accuracy of our antigen-specific immunoassays is suboptimal for clinical laboratory applications, this study provides a simultaneous and detailed multiparameter immunoprofiling characterization of cytokine/activation T-cell markers in IGRA/TST−negative exposed individuals. Our study results support prior proof-of-concept studies characterizing this important, but understudied group of TB-exposed individuals (Morrison et al., 2008; Simmons et al., 2018; Shey et al., 2023; Mubeen et al., 2025). This study and others also highlight the limited understanding of early/low-grade Mtb infection biology and initial host responses in humans.

Our study has several limitations. TST results were retrospectively obtained from electronic medical records, and TST data were unavailable for 33.3% of total participants, which probably reflects a shift in practice toward the preferred use of IGRA over TST for most adult patients across our practices in our center. Nevertheless, all study subjects had negative IGRA results and no clinical, radiological, or microbiological evidence of active tuberculosis, which supports the overall validity of the cohort classification. As in many immunoprofiling studies in individuals at risk of Mtb infection, precise Mtb exposure classification is difficult, and undetected exposure in controls or variable exposure timing in TB-exposed individuals cannot be excluded. Exposure timing was actively determined by study questionnaires (**Table 1**), but retrospective estimation of timing and degree of Mtb exposures rely on self-report and indirect indicators (i.e. residence in TB endemic areas). Exposure timing and intensity are important for immune profiling studies, as they influence T-cell activation markers like HLA-DR and cytokine expression. Pooling heterogeneous exposure and timing can introduce biological variability to our study data, though individual infectivity varies widely, as demonstrated in classic transmission studies using guinea pigs as sensitive surrogates of infection (Sultan et al., 1960). Another limitation is that most Mtb-exposed participants were HCW which may have introduced occupational exposures that contributed to some of the observed immune responses, including responses to Candida antigens.

Prior immunoprofiling studies support analyzing overall TB exposure history despite imperfect timing data. Risk stratification by IGRA conversion versus persistence-IGRA positivity as surrogate of recent vs. remote Mtb infection, reveals Mtb-specific immune differences without precise Mtb exposure details in an high endemic TB area (Mpande et al., 2021a). Our study was conducted in a low-endemic TB setting, so our TB-unexposed controls are very likely Mtb-uninfected and thus the possibilities of potential confounders associated with casual or inadvertent TB exposures are minimal. The results of our subgroup analysis comparing immunoprofiling results between recent (n = 8) versus remote (n = 5) TB exposure was interesting; however, given the small subgroup sizes and wide variability, this study finding should be interpreted cautiously as exploratory and are not sufficient to draw firm conclusions about historical time−dependent effects. Of note, other important immunoprofiling studies that generated significant insights utilized comparable sample sizes (Wilkinson et al., 2009; Nyendak et al., 2013; Escalante et al., 2015). Single-time-point IGRA testing may have missed dynamic testing conversions, though unlikely in individuals without recent TB exposure. Although flow cytometry immunoprofiling provides detailed T-cell characterizations, its complexity limits routine laboratory implementation.

## 5 Conclusions

Taking together, our data supports that flow cytometric functional T cell assays target IFN-γ production and HLA-DR expression following mycobacterial antigen stimulations as sensitive tools for identifying individuals with potential Mtb exposure. Prospective studies with careful determination of timing and degree of Mtb exposures would be needed to determine whether antigen-specific IFN-γ levels in antigen-stimulated HLA-DR+ T-cells can also detect false negative IGRA results in individuals Mtb infection after exposures in addition to the potential value to detect IGRA-positive individuals at increased risk to progressing to active TB. We also propose that these immunoprofiling methods be further evaluated for their utility in risk stratification in Mtb infections and for selecting unexposed donors in future vaccine trials and TB prevention studies.

## Data Availability

The original contributions presented in the study are included in the article/**Supplementary Material**. Further inquiries can be directed to the corresponding author.
