# Supplementary material for: Multiparameter antigen-specific immunoprofiling in subjects with negative IGRA and TST results with potential M. tuberculosis exposures
Source: Front Cell Infect Microbiol. 2026 May 1;16:1837269. doi: 10.3389/fcimb.2026.1837269 (PMC13176205; doi:10.3389/fcimb.2026.1837269)
Supplement: Supplementary file 1 [file DataSheet1.pdf]

Supplementary Figure 1. Gating strategy followed for assessing functional characterization of T-cells

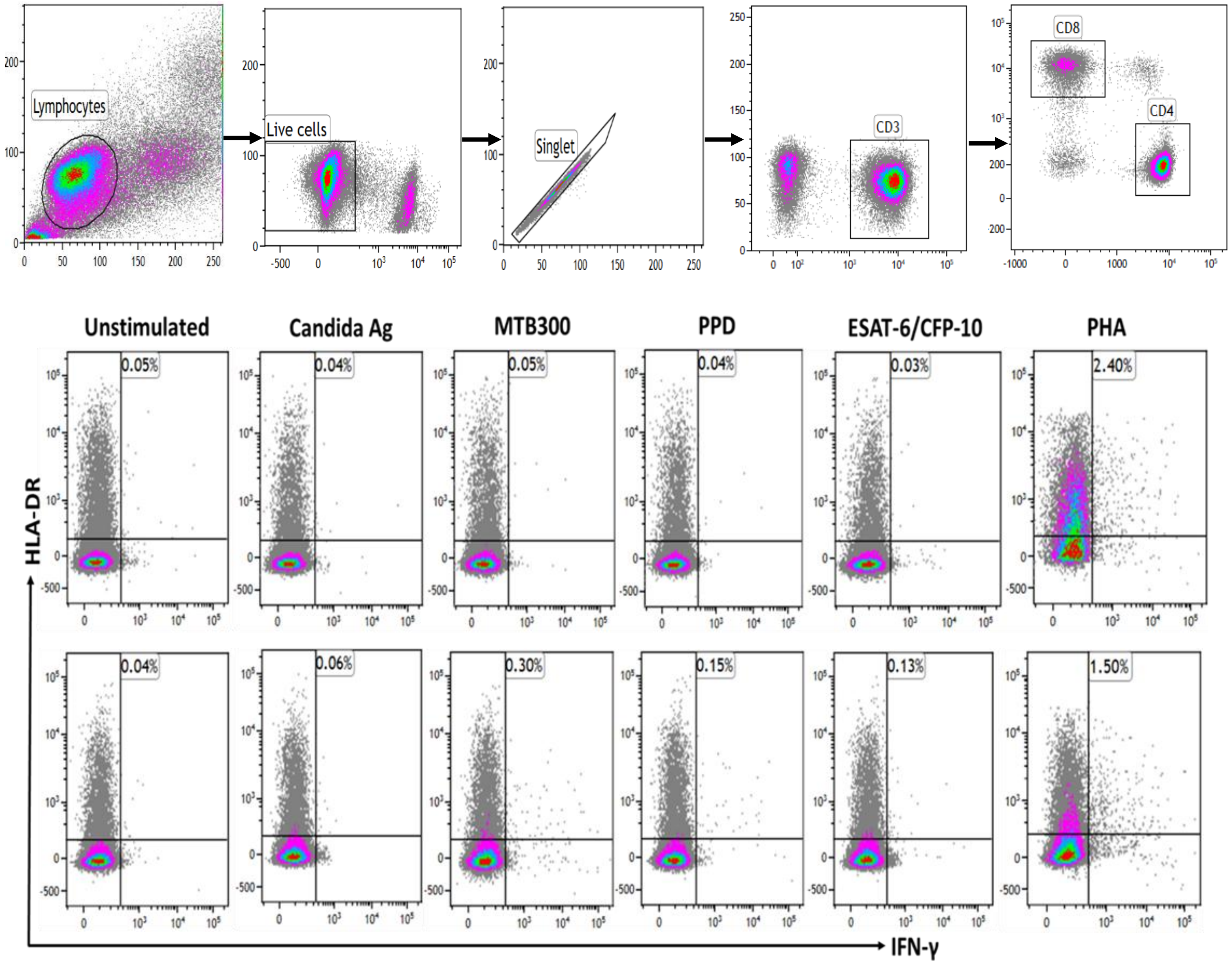

Gating was performed on single, live lymphocytes, followed by selection of $CD3^+$ T cells and subsequent identification of $CD4^+$ and $CD8^+$ T cell subsets. Functional and activation markers (HLA-DR, IFN-γ, TNF-α, CD25, CD134, PD-L1) were analyzed within these populations. Representative dot plots are gated on $CD4^+$ T cells, displaying IFN-γ expression on the x-axis and HLA-DR on the y-axis for antigen-stimulated samples. The upper panel depicts an unexposed individual, while the lower panel shows a TB-exposed individual. The value in the upper right quadrant indicates the frequency of $CD4^+$ T cells co-expressing HLA-DR and IFN-γ. Data were analyzed using Kaluza Analysis software.
