# Supplementary material for: Multiparameter antigen-specific immunoprofiling in subjects with negative IGRA and TST results with potential M. tuberculosis exposures
Source: Front Cell Infect Microbiol. 2026 May 1;16:1837269. doi: 10.3389/fcimb.2026.1837269 (PMC13176205; doi:10.3389/fcimb.2026.1837269)
Supplement: Supplementary file 2 [file DataSheet2.pdf]

Supplementary Figure 2: Immunophenotyping results of FC assays in recent and remote TB exposure group

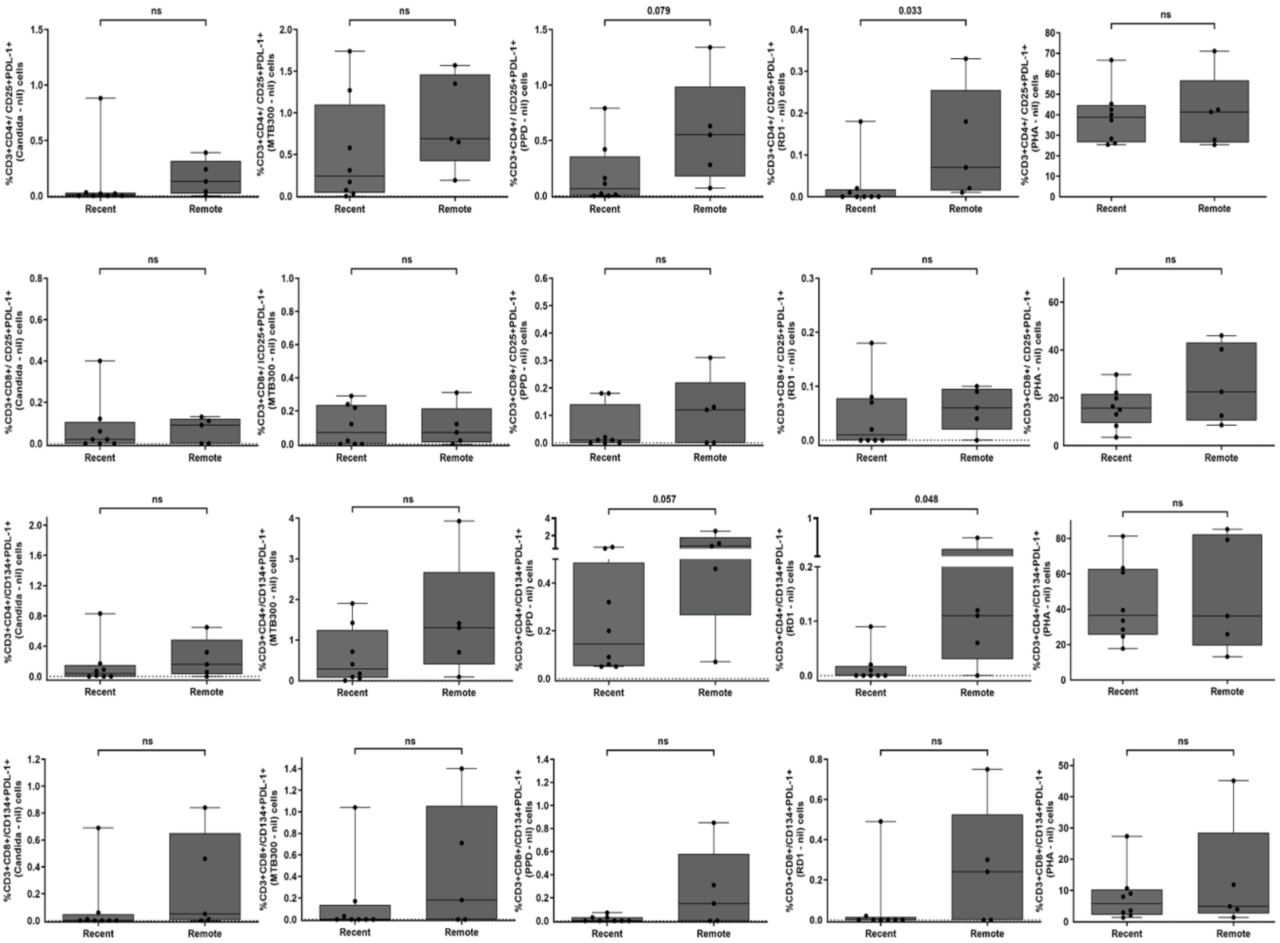

Flow cytometric detection of the percentage of $CD3^{+}CD4^{+}CD8^{+}$ T cells co-expressing $CD25^{+}PDL\text{-}1^{+}$ and $CD134^{+}PD\text{-}L1^{+}$ following stimulation with Candida antigen, MTB300, PPD, RD1 peptides, and PHA. For each donor, responses to stimulated cells were background-subtracted. Group differences were analyzed using the Mann–Whitney U-test. The horizontal line indicates the median; the upper and lower boundaries of each box represent the 75th and 25th percentiles, respectively. Whiskers extend to the minimum and maximum values. ns = not significant ($P \geq 0.05$).
