# Supplementary material for: Multiparameter antigen-specific immunoprofiling in subjects with negative IGRA and TST results with potential M. tuberculosis exposures
Source: Front Cell Infect Microbiol. 2026 May 1;16:1837269. doi: 10.3389/fcimb.2026.1837269 (PMC13176205; doi:10.3389/fcimb.2026.1837269)
Supplement: Supplementary file 3 [file DataSheet3.pdf]

Supplementary Figure 3: Ratio of activated antigen-specific cells ($CD4^+/CD8^+IFN\text{-}\gamma^+HLA\text{-}DR^+$) to total antigen-specific cells

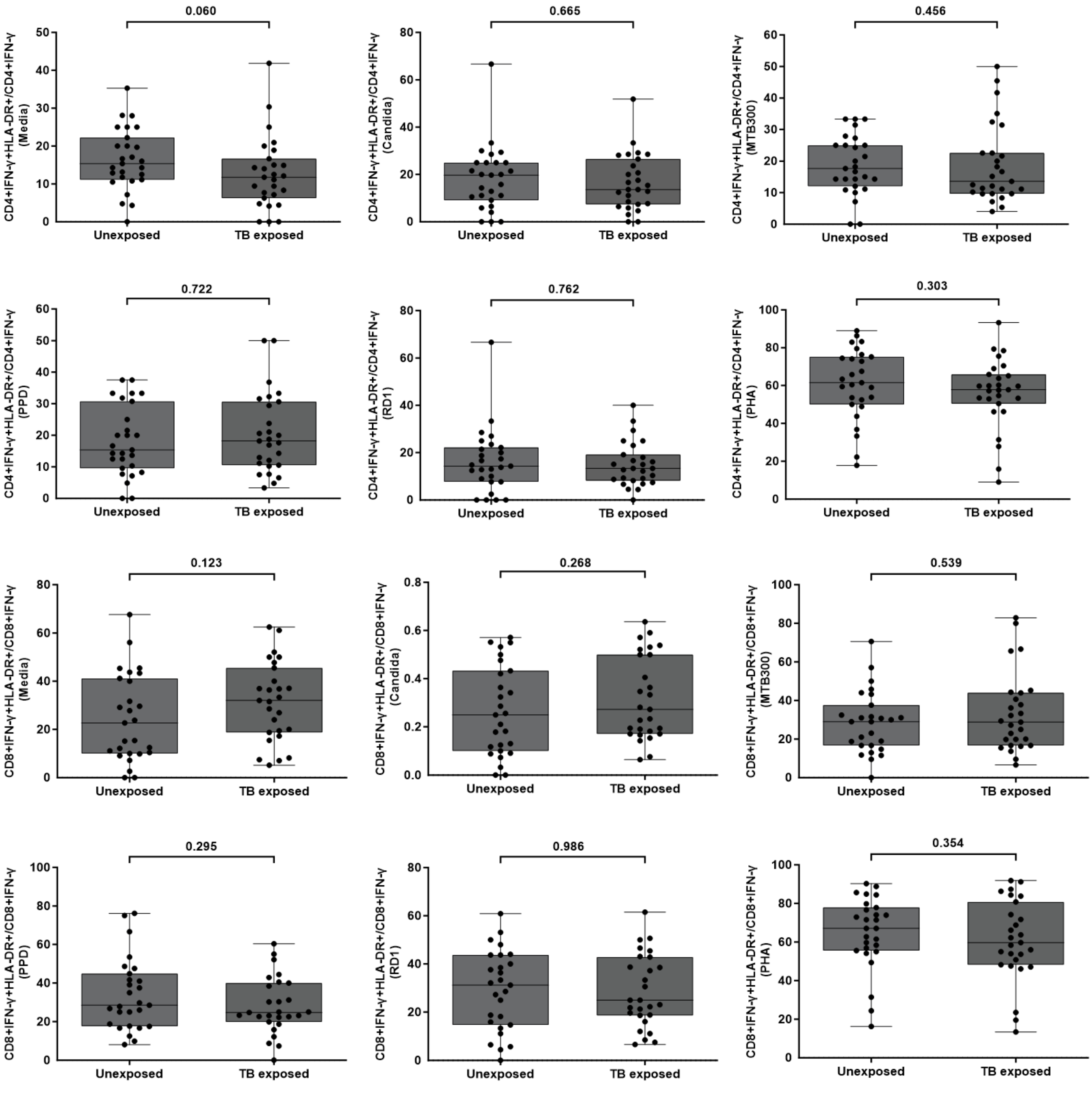

The data was normalized by calculating the ratio of activated antigen-specific cells ($CD4^+/CD8^+IFN\text{-}\gamma^+HLA\text{-}DR^+$) to total antigen-specific cells ($CD4^+/CD8^+IFN\text{-}\gamma^+$) following stimulation with Candida antigen, MTB300, PPD, RD1 peptides, and PHA. Group differences were analyzed using the Mann–Whitney U-test. The horizontal line indicates the median; the upper and lower boundaries of each box represent the 75th and 25th percentiles, respectively. Whiskers extend to the minimum and maximum values.
