# Supplementary material for: Multiparameter antigen-specific immunoprofiling in subjects with negative IGRA and TST results with potential M. tuberculosis exposures
Source: Front Cell Infect Microbiol. 2026 May 1;16:1837269. doi: 10.3389/fcimb.2026.1837269 (PMC13176205; doi:10.3389/fcimb.2026.1837269)
Supplement: Supplementary file 4 [file DataSheet4.pdf]

Supplementary Table 1: Mean and median proportions and interquartile ranges of QFT and FC assays in unexposed and exposed controls

Immunodiagnostic Assays	TB Unexposed (n =27)<br>Mean ± SD<br>Median (IQR)	TB Exposed (n = 27)<br>Mean ± SD<br>Median (IQR)
QFT plus (tube 1-nil) IU/ml	0.025 ± 0.054<br>0.0 (0 – 0.21)	0.032 ± 0.073<br>0.00 (0 – 0.33)
QFT plus, (tube 2-nil) IU/ml	0.028 ± 0.046<br>0.01 (0 – 0.21)	0.038 ± 0.086<br>0.01 (0 – 0.33)
%$CD3^+CD4^+$/ IFN-g^+TNF-a^+ (Candida - nil)	0.011 ± 0.026<br>0 (0 – 0.11)	0.027 ± 0.052<br>0 (0 – 0.25)
%$CD3^+CD4^+$/ IFN-g^+TNF-a^+ (MTB300 - nil	0.013 ± 0.018<br>0.0 (0 – 0.06)	0.027 ± 0.055<br>0 (0 – 0.27)
%$CD3^+CD4^+$/ IFN-g^+TNF-a^+ (PPD - nil)	0.007 ± 0.012<br>0.0 (0 – 0.04)	0.020 ± 0.038<br>0 (0 – 0.15)
%$CD3^+CD4^+$/ IFN-g^+TNF-a^+ (RD1 - nil)	0.010 ± 0.02<br>0.0 (0 – 0.07)	0.015 ± 0.024<br>0 (0 – 0.10)
%$CD3^+CD4^+$/ IFN-g^+TNF-a^+ (PHA - nil)	0.686 ± 0.657<br>0.46 (0.02 – 2.65)	0.897 ± 0.962<br>0.58 (0 – 4.27)
%$CD3^+CD8^+$/ IFN-g^+TNF-a^+ (Candida - nil)	0.028 ± 0.058<br>0 (0 – 0.22)	0.052 ± 0.146<br>0 (0 – 0.74)
%$CD3^+CD8^+$/ IFN-g^+TNF-a^+ (MTB300 - nil)	0.05 ± 0.16<br>0 (0 – 0.81)	0.094 ± 0.191<br>0 (0 – 0.73)
%$CD3^+CD8^+$/ IFN-g^+TNF-a^+ (PPD - nil)	0.03 ± 0.082<br>0 (0 – 0.42)	0.025 ± 0.071<br>0 (0 – 0.36)
%$CD3^+CD8^+$/ IFN-g^+TNF-a^+ (RD1 - nil)	0.01 ± 0.021<br>0 (0 – 0.08)	0.121 ± 0.309<br>0 (0 – 1.53)
%$CD3^+CD8^+$/ IFN-g^+TNF-a^+ (PHA - nil)	1.01 ± 1.23<br>0.64 (0.03 – 6.23)	1.679 ± 1.925<br>1.41 (0 – 9.05)
%$CD3^+CD4^+$/ IFN-g^+HLADR$^+$ (Candida - nil)	0.003 ± 0.006<br>0 (0 – 0.02)	0.016 ± 0.023<br>0.01 (0 – 0.08)
%$CD3^+CD4^+$/ IFN-g^+HLADR$^+$ (MTB300 - nil)	0.016 ± 0.027<br>0 (0 – 0.12)	0.037 ± 0.047<br>0.03 (0 – 0.23)
%$CD3^+CD4^+$/ IFN-g^+HLADR$^+$ (PPD - nil)	0.007 ± 0.014<br>0 (0 – 0.07)	0.03 ± 0.039<br>0.01 (0 – 0.14)
%$CD3^+CD4^+$/ IFN-g^+HLADR$^+$ (RD1 - nil)	0.009 ± 0.022<br>0 (0 – 0.10)	0.032 ± 0.041<br>0.02 (0 – 0.19)
%$CD3^+CD4^+$/ IFN-g^+HLADR$^+$ (PHA - nil)	1.88 ± 1.93<br>1.25 (0.04 – 7.53)	2.715 ± 3.571<br>1.73 (0.18 – 19.12)
%$CD3^+CD8^+$/ IFN-g^+HLADR$^+$ (Candida - nil)	0.01 ± 0.017<br>0 (0 – 0.06)	0.03 ± 0.054<br>0 (0 – 0.24)
%$CD3^+CD8^+$/ IFN-g^+HLADR$^+$ (MTB300 - nil)	0.048 ± 0.107<br>0.02 (0 – 0.56)	0.094 ± 0.187<br>0.03 (0 – 0.91)
%$CD3^+CD8^+$/ IFN-g^+HLADR$^+$ (PPD - nil)	0.040 ± 0.072<br>0.02 (0 – 0.30)	0.038 ± 0.056<br>0 (0 – 0.17)
%$CD3^+CD8^+$/ IFN-g^+HLADR$^+$ (RD1 - nil)	0.025 ± 0.028	0.025 ± 0.028

	0.02 (0 – 0.10)	0.07 (0 – 0.50)
%CD3⁺CD8⁺/ IFN-g⁺HLADR⁺ (PHA - nil)	4.69 ± 4.35<br>3.02 (0.08 – 16.43)	5.656 ± 4.046<br>4.38 (0.74 – 13.83)
%CD3⁺CD4⁺/ TNF-a⁺HLADR⁺ (Candida - nil)	0.016 ± 0.031<br>0 (0 – 0.12)	0.015 ± 0.020<br>0 (0 – 0.07)
%CD3⁺CD4⁺/ TNF-a⁺HLADR⁺ (MTB300 - nil)	0.003 ± 0.007<br>0 (0 – 0.03)	0.017 ± 0.038<br>0 (0 – 0.10)
%CD3⁺CD4⁺/ TNF-a⁺HLADR⁺ (PPD - nil)	0.012 ± 0.02<br>0 (0 – 0.08)	0.021 ± 0.038<br>0 (0 – 0.15)
%CD3⁺CD4⁺/ TNF-a⁺HLADR⁺ (RD1 - nil)	0.008 ± 0.017<br>0 (0 – 0.06)	0.018 ± 0.033<br>0 (0 – 0.12)
%CD3⁺CD4⁺/ TNF-a⁺HLADR⁺ (PHA - nil)	3.51 ± 3.01<br>2.66 (0.04 – 14.48)	3.66 ± 2.355<br>3.33 (0.94 – 10.49)
%CD3⁺CD8⁺/ TNF-a⁺HLADR⁺ (Candida - nil)	0.031 ± 0.056<br>0 (0 – 0.20)	0.034 ± 0.055<br>0 (0 – 0.22)
%CD3⁺CD8⁺/ TNF-a⁺HLADR⁺ (MTB300 - nil)	0.025 ± 0.063<br>0 (0 – 0.32)	0.06 ± 0.102<br>0 (0 – 0.31)
%CD3⁺CD8⁺/ TNF-a⁺HLADR⁺ (PPD - nil)	0.050 ± 0.058<br>0.05 (0 – 0.22)	0.029 ± 0.062<br>0 (0 – 0.30)
%CD3⁺CD8⁺/ TNF-a⁺HLADR⁺ (RD1 - nil)	0.027 ± 0.046<br>0 (0 – 0.21)	0.029 ± 0.062<br>0 (0 – 0.23)
%CD3⁺CD8⁺/ TNF-a⁺HLADR⁺ (PHA - nil)	1.50 ± 2.01<br>1.12 (0.09 – 4.84)	2.01 ± 1.589<br>2.04 (0.26 – 6.84)
%CD3⁺CD4⁺/ CD25⁺CD134⁺ (Candida - nil)	0.043 ± 0.063<br>0.01 (0 – 0.22)	0.13 ± 0.288<br>0.02 (0 – 1.14)
%CD3⁺CD4⁺/ CD25⁺CD134⁺ (MTB300 - nil)	0.304 ± 0.249<br>0.21 (0 – 0.83)	0.502 ± 0.60<br>0.310 (0 – 2.39)
%CD3⁺CD4⁺/ CD25⁺CD134⁺ (PPD - nil)	0.144 ± 0.194<br>0.05 (0 – 0.69)	0.267 ± 0.324<br>0.15 (0 – 1.11)
%CD3⁺CD4⁺/ CD25⁺CD134⁺ (RD1 - nil)	0.002 ± 0.007<br>0 (0 – 0.03)	0. 044 ± 0.083<br>0 (0 – 0.29)
%CD3⁺CD4⁺/ CD25⁺CD134⁺ (PHA - nil)	26.07 ± 16.51<br>25.4 (1.7 – 55.42)	30.66 ± 14.44<br>28.46 (10.79 – 66.47)
%CD3⁺CD8⁺/ CD25⁺CD134⁺ (Candida - nil)	0.025 ± 0.047<br>0 (0 – 0.21)	0.064 ± 0.095<br>0.01 (0 – 0.35)
%CD3⁺CD8⁺/ CD25⁺CD134⁺ (MTB300 - nil	0.065 ± 0.108<br>0.01 (0 – 0.34)	0.074 ± 0.097<br>0.004 (0 – 0.37)
%CD3⁺CD8⁺/ CD25⁺CD134⁺ (PPD - nil)	0.072 ± 0.156<br>0.02 (0 – 0.73)	0.075 ± 0.132<br>0.01 (0 – 0.47)
%CD3⁺CD8⁺/ CD25⁺CD134⁺ (RD1 - nil)	0.022 ± 0.037<br>0 (0 – 0.12)	0.039 ± 0.063<br>0 (0 – 0.22)
%CD3⁺CD8⁺/ CD25⁺CD134⁺ (PHA - nil)	9.94 ± 11.62<br>5.6 (0.55 – 46.42)	11.62 ± 12.06<br>8.54 (1.18 – 55.69)
%CD3⁺CD4⁺/ CD25⁺PDL1⁺ (Candida - nil)	0.049 ± 0.097<br>0.02 (0 – 0.48)	0.125 ± 0.277<br>0.02 (0 – 1.17)
%CD3⁺CD4⁺/ CD25⁺PDL1⁺ (MTB300 - nil)	0.33 ± 0.311<br>0.24 (0 – 1.11)	0.539 ± 0.564<br>0.29 (0 – 1.75)

%CD3$^+$CD4$^+$/ CD25$^+$PDL1$^+$ (PPD - nil)	0.173 ± 0.251<br>0.075 (0 – 0.86)	0.263 ± 0.333<br>0.16 (0 – 1.34)
%CD3$^+$CD4$^+$/ CD25$^+$PDL1$^+$ (RD1 - nil)	0.027 ± 0.066<br>0 (0 – 0.32)	0.048 ± 0.085<br>0.01 (0 – 0.33)
%CD3$^+$CD4$^+$/ CD25$^+$PDL1$^+$ (PHA - nil)	34.46 ± 17.42<br>36.29 (2.28 – 65.36)	37.78 ± 14.24<br>36.94 (14.6 – 71.09)
%CD3$^+$CD8$^+$/ CD25$^+$PDL1$^+$ (Candida - nil)	0.067 ± 0.171<br>0 (0 – 0.71)	0.07 ± 0.096<br>0.02 (0 – 0.4)
%CD3$^+$CD8$^+$/ CD25$^+$PDL1$^+$ (MTB300 - nil)	0.088 ± 0.182<br>0.02 (0 – 0.94)	0.107 ± 0.141<br>0.05 (0 – 0.56)
%CD3$^+$CD8$^+$/ CD25$^+$PDL1$^+$ (PPD - nil)	0.064 ± 0.122<br>0.01 (0 – 0.48)	0.069 ± 0.107<br>0.01 (0 – 0.40)
%CD3$^+$CD8$^+$/ CD25$^+$PDL1$^+$ (RD1 - nil)	0.036 ± 0.045<br>0 (0 – 0.13)	0.036 ± 0.058<br>0 (0 – 0.22)
%CD3$^+$CD8$^+$/ CD25$^+$PDL1$^+$ (PHA - nil)	20. 58 ± 15.41<br>17.06 (0.67 – 52.37)	17.46 ± 11.81<br>15.07 (3.15 – 46.02)
%CD3$^+$CD4$^+$/ CD134$^+$PDL1$^+$ (Candida - nil)	0.131 ± 0.391<br>0.0 (0 – 2.04)	0.142 ± 0.228<br>0.005 (0 – 0.83)
%CD3$^+$CD4$^+$/ CD134$^+$PDL1$^+$ (MTB300 - nil)	0.45 ± 0.636<br>0.21 (0 – 2.73)	0.107 ± 0.141<br>0.27 (0 – 3.93)
%CD3$^+$CD4$^+$/ CD134$^+$PDL1$^+$ (PPD – nil)	0.338 ± 0.630<br>0.12 (0 – 3.02)	0.374 ± 0.520<br>0.2 (0 – 2.53)
%CD3$^+$CD4$^+$/ CD134$^+$PDL1$^+$ (RD1 - nil)	0.025 ± 0.115<br>0 (0 – 0.6)	0.042 ± 0.115<br>0 (0 – 0.59)
%CD3$^+$CD4$^+$/ CD134$^+$PDL1$^+$ (PHA - nil)	40.21 ± 23.07<br>39.58 (1.98 – 86.1)	42.97 ± 20.94<br>36.85 (13.21 – 85.23)
%CD3$^+$CD8$^+$/ CD134$^+$PDL1$^+$ (Candida - nil)	0.06 ± 0.19<br>0 (0 – 0.98)	0.125 ± 0.235<br>0.01 (0 – 0.84)
%CD3$^+$CD8$^+$/ CD134$^+$PDL1$^+$ (MTB300 - nil)	0.085 ± 0.126<br>0.02 (0 – 0.41)	0.150 ± 0.346<br>0 (0 – 1.4)
%CD3$^+$CD8$^+$/ CD134$^+$PDL1$^+$ (PPD - nil)	0.137 ± 0.326<br>0 (0 – 1.37)	0.064 ± 0.173<br>0 (0 – 0.85)
%CD3$^+$CD8$^+$/ CD134$^+$PDL1$^+$ (RD1- nil)	0.123 ± 0.189<br>0.02 (0 – 0.65)	0.081 ± 0.181<br>0 (0 – 0.75)
%CD3$^+$CD8$^+$/ CD134$^+$PDL1$^+$ (PHA - nil)	7.25 ± 9.96<br>2.97 (0.33 – 44.11)	8.021 ± 9.432<br>5.95 (0.48 – 45.15)
